# Treosulfan plus fludarabine versus TEAM as conditioning treatment before autologous stem cell transplantation for B-cell Non-Hodgkin lymphoma

**DOI:** 10.1038/s41409-022-01701-x

**Published:** 2022-05-10

**Authors:** Jochen J. Frietsch, Jenny Miethke, Paul Linke, Carl C. Crodel, Ulf Schnetzke, Sebastian Scholl, Andreas Hochhaus, Inken Hilgendorf

**Affiliations:** Klinik für Innere Medizin II, Hämatologie und internistische Onkologie, Universitätsklinikum Jena, Jena, Germany

**Keywords:** Medical research, B-cell lymphoma

## Abstract

Conditioning with treosulfan and fludarabine (Treo/Flu) has been proven to be feasible and efficient in several types of malignancies before allogeneic hematopoietic stem cell transplantation (allo-HSCT). Given its favorable reduced toxicity profile, we introduced Treo/Flu as conditioning before autologous HSCT (auto-HSCT) in patients with B-cell Non-Hodgkin lymphoma (NHL). The aim of this study was to evaluate the efficacy and safety of Treo/Flu in comparison to TEAM. Fifty-seven patients with NHL received auto-HSCT after conditioning with either Treo/Flu (*n* = 22) or TEAM (*n* = 35). All patients achieved sustained engraftment. PFS, EFS and OS were not significant in both groups. Of note is that patients in the Treo/Flu group were less dependent on thrombocyte transfusions (*p* = 0.0082), significantly older (in median 11 years, *p* < 0.0001) and suffered less frequently from infectious complications (*p* = 0.0105), mucositis and stomatitis (*p* < 0.0001). This study is the first to present efficacy, feasibility, and safety of conditioning with Treo/Flu preceding auto-HSCT in patients with NHL. Since it demonstrated a lack of significant difference in comparison to TEAM conditioning it might be a valuable alternative especially in elderly patients with B-cell NHL and comorbidities. Further evaluation by prospective clinical trials is warranted.

## Introduction

Non-Hodgkin lymphoma (NHL) represents a heterogeneous group from indolent to the most aggressive malignancies [1]. Despite the improvement of therapeutic options, up to 30 to 40% of patients with B-cell NHL may experience relapse or refractoriness [2]. Nevertheless, high-dose chemotherapy (HDT) followed by autologous hematopoietic stem cell transplantation (auto-HSCT) can induce remission in those cases of relapsed or refractory (R/R) lymphoma [3]. As it is also associated with prolonged survival rates, auto-HSCT became further an integral part of the primary management of mantle cell lymphoma (MCL) [4, 5].

One of the most commonly used HDT regimen is the BEAM (BCNU/carmustine, etoposide, cytarabine, and melphalan) protocol [6–8]. However, BCNU/carmustine is associated with a number of toxicities, e.g., pulmonary side effects. In addition, disposability and cost issues for carmustine promoted the replacement of BCNU/carmustine with thiotepa. Subsequently, conditioning with TEAM was considered as a valuable alternative to BEAM in auto-HSCT for lymphoma [9].

However, the age of patients undergoing auto-HSCT and, therefore, the comorbidities, have increased over the last decades [10]. Several years ago, the combination of the alkylating agent treosulfan with the nucleoside analogue fludarabine (Treo/Flu) has been successfully introduced as reduced toxicity conditioning (RTC) before allo-HSCT [11–14]. Recently, the non-inferiority of Treo/Flu in comparison to busulfan and fludarabine as conditioning treatment before allo-HSCT for older patients with acute myeloid leukemia or myelodysplastic syndrome could be demonstrated in a phase three trial [15]. In addition, improved overall survival (OS) in first-line HSCT after Treo/Flu based conditioning was reported by a large retrospective analysis of the chronic malignancy working party of the European Society for Blood and Marrow Transplantation (EBMT) [16]. The feasibility of high-dose treosulfan as major therapy component in patients with relapsed high-grade lymphoma resulting in sustained complete remissions (CR) after auto-HSCT was reported by Koenigsmann et al. [17]. In a prospective, risk-adapted, multicenter phase II trial (Trial 071), the East German Study Group for Hematology and Oncology (OSHO) evaluated treosulfan as part of a HDT regimen for both auto- and allo-HSCT in patients with R/R aggressive NHL [18]. Briefly, all patients received auto-HSCT after conditioning with (R)-TEC (rituximab, treosulfan, etoposide, carboplatin) followed by either no further treatment, a second course of R-TEC or allo-HSCT after conditioning with Treo/Flu resulting in a response rate of 53%. Further encouraged by the results of Yerushalmi et al. [19] and Schmitt et al. [20] who showed effectiveness and more balanced outcomes after conditioning with Treo/Flu preceding allo-HSCT for lymphoma, we evaluated Treo/Flu as conditioning therapy followed by auto-HSCT for elderly patients with B-cell NHL not eligible for intensive conditioning with TEAM. The aim of this retrospective study is to evaluate its efficacy and feasibility in comparison to myeloablative conditioning with TEAM.

## Materials, patients, and methods

### Data source, patient selection, and scoring

The internal clinical database was searched for recipients of auto-HSCT after conditioning with TEAM, or Treo/Flu for diffuse large B-cell lymphoma (DLBCL), follicular lymphoma (FL), or MCL. Survival was estimated according to IPI, FLIPI, and MIPI scores [21–23] and relevant comorbidities and HSCT associated risks were additionally identified using the hematopoietic cell transplantation-specific comorbidity index (HCT-CI) [24]. A retrospective review of individual medical records was performed. Written informed consent for HDT, auto-HSCT, data collection and analysis were obtained from all patients in this report.

### Transplantation procedures

Between 2012 and 2021, a total of 57 recipients of auto-HSCT for the treatment of B-cell NHL were included into this retrospective analysis. The patient characteristics are summarized in Table 1. The majority of patients (35/57; 61.4%) received myeloablative conditioning (MAC) therapy according to the TEAM (thiotepa, 5 mg/kg body weight twice daily at day −6; cytarabine, 2 × 200 mg/m² per day from day −6 to day −3; etoposide, 2 × 150–200 mg/m² per day from day −6 to day −3; and melphalan, 140 mg/m² at day −2) protocol irrespectively of the addition of the humanized monoclonal CD20 antibody rituximab. Dosage reduction was performed based on individual patient specific factors. The remaining patients (22/58; 38.6%) received RTC with Treo/Flu adapted from the allogenic setting (treosulfan, 14,000 mg/m² from day −4 to day −2 and fludarabine, 30 mg/m² from day −6 to day −2) [25] prior to auto-HSCT. Allocation of patients to conditioning protocol was based on attending physician’s choice and on the patient’s comorbidities. Chemotherapy- (cyclophosphamide) and granulocyte colony-stimulating factor (G-CSF)–mobilized peripheral blood stem cell grafts were used in all cases [26].

**Table 1:** Characteristics of all patients according to HDT.

Patient characteristics	TEAM (*n* = 35)	Treo/Flu (*n* = 22)	*P*-value
Median follow-up [days] (range)	587 (66–2905)	674 (91–1777)	0.7729^■^
Median age at HSCT [years] (range)	54 (23–63)	65 (49–73)	**<0.0001**^■^
Median disease stage at HSCT [30]	IV (I–IV)	IV (I–IV)	0.2654^■^
Gender			0.2769^▼^
Male	23 (65.7%)	11 (50.0%)	
Female	12 (34.3%)	11 (50.0%)	
Disease status at HSCT			
CR	8 (22.9%)	4 (18.2%)	0.7496^▼^
PR	23 (65.7%)	16 (72.7%)	(CR vs. non-CR)
PD	4 (11.4%)	2 (9.1%)	
Number of treatment courses before HSCT			
Median	3	2	
One	0 (0%)	0 (0%)	0.2640^■^
Two	15 (42.9%)	13 (59.1%)	
Three	16 (45.7%)	7 (31.8%)	
Four	2 (5.7%)	2 (9.1%)	
Five	1 (2.9%)	0 (0%)	
Six	1 (2.9%)	0 (0%)	
Disease			
DLBCL	23 (65.7%)	13 (59.1%)	0.7786^▼^
FL	3 (8.6%)	2 (9.1%)	(HML vs. LML)
MCL	9 (25.7%)	7 (31.8%)	
IPI at HSCT (number of patients with available IPI) [21–23]	1/27 low ‡	1/15 low ‡	
	2/27 intermediate ‡	3/15 intermediate ‡	
	2/27 high ‡	1/15 high ‡	
	1/27 intermediate †	1/15 intermediate †*	
	1/27 intermediate †*		
	1/27 high †		
	3/27 high †*		
	6/27 low^♦^	3/15 low-intermediate^♦^	
	5/27 low-intermediate^♦^	3/15 high-intermediate^♦^	
	1/27 low-intermediate^♦^*	1/15 high-intermediate^♦^*	
	2/27 high-intermediate^♦^	2/15 high^♦^	
	2/27 high^♦^		
Median HCT-CI (range) [24]	0 (0–7)	1 (0–6)	0.1499^■^
Year of transplantation			
2013	3 (8.3%)	0 (0%)	
2014	12 (33.3%)	0 (0%)	
2015	1 (2.8%)	0 (0%)	
2016	4 (11.1%)	4 (18.2%)	
2017	2 (5.6%)	4 (18.2%)	
2018	3 (8.3%)	3 (13.6%)	
2019	3 (8.3%)	4 (18.2%)	
2020	8 (22.2%)	5 (22.7%)	
2021	0 (0%)	2 (9.1%)	
Median number of infused CD34 positive stem cells [x 10E6/kg body weight] (range)	2.59 (2.01–7.10)	2.73 (2.04–7.12)	0.8613^■^
Median number of transfused erythrocyte/thrombocyte concentrates (range)	5 (0–18)/5 (2-30)	4 (0–12)/4 (0-11)	0.1263^■^/**0.0082**^■^
Median time of engraftment of leukocytes/thrombocytes [days] (range)	10 (9–16)/19 (14–83)	10 (8–16)/18 (10–32)	**0.0197**^■^/0.0979^■^
Median period of hospitalization following reinfusion of stem cells [days] (range)	20 (13–52)	17.5 (13–35)	**0.0484**^■^

*CR* Complete remission, *DLBCL* Diffuse large B-cell lymphoma, *FL* Follicular lymphoma, *HCT-CI* Hematopoietic cell transplantation-specific comorbidity index, *HDT* High-dose chemotherapy, *HML* High-malignant lymphoma, *HSCT* Hematopoietic stem cell transplantation, *LML* Low-malignant lymphoma, *MCL* Mantle cell lymphoma, *NHL* Non-Hodgkin lymphoma, *PD* Progressive disease, *PR* Partial response, ^♦^IPI, †FLIPI, ‡MIPI, *transformation to secondary high-malignant lymphomas, ^■^ Mann–Whitney *U* test; ^▼^Fisher’s exact probability test, figures in bold indicate significant *p*-values.

### Definition of engraftment and blood support

Leukocyte engraftment was defined as the first of 3 consecutive days of an absolute neutrophil count of ≥0.5 × 10^9^/l [27]. Engraftment of platelets was defined as the first of 3 consecutive days of increasing platelet count above 20 × 10^9^/l without transfusion support for 7 days [28]. Red cell and platelet transfusions were given to maintain hemoglobin levels higher than 80 g/L and platelet counts higher than 10 × 10^9^/L.

### Adverse effects and outcome evaluation

Progression free survival (PFS) was calculated from the date of reinfusion of hematopoietic stem cells to date of disease progression as detected by imaging techniques. The date of disease progression, conduct of another cell therapy, solid organ transplantation, diagnosis of another cancerous disease or death due to any cause lead to the calculation of the event free survival (EFS). Overall survival (OS) was calculated from the date of reinfusion of hematopoietic stem cells to date of death. Non-relapse mortality (NRM) was defined as any death without recurrent lymphoma. Toxicities and adverse events as defined by the common terminology criteria for adverse events (CTCAE, NCI, Bethesda, MD, USA) version 5.0 were recorded during hospitalization.

### Statistical analysis

Differences in engraftment after different HDT and comparison of variables between groups were analyzed using Mann–Whitney *U* test as indicated. Fisher’s exact probability test was used in the adverse event, gender, disease, and disease status comparison. Differences between the Kaplan–Meier survival plots were evaluated by Log-rank test. A *P*-value of <0.05 was considered statistically significant. All analyses were conducted using GraphPad Prism 9.3.1 (GraphPad Inc.) except cumulative incidence curves with competing risk analysis, which have been performed with Gray’s test using R, version 4.1.2 provided by the R Foundation [29].

## Results

### Patients’ characteristics

Between January 1st, 2012, and June 30st, 2021, a total of 57 patients with B-cell NHL were included in this analysis, of whom 35 (61.4%) received TEAM and 22 (38.6%) Treo/Flu conditioning. The patients suffered from either DLBCL, FL, or MCL as follows: 36/57 (63.2%), 5/57 (8.8%) and 16/57 (28.1%), respectively. The allocation of disease subgroups to the particular conditioning therapy can be selected from Table 1. DLBCL, FL and MCL were all classified at diagnosis with an median disease stage of III, IV, and IV, respectively, according to Ann Arbor staging classification [30]. However, calculation of IPI, FLIPI, and MIPI scores was possible in 42/57 (73.7%) cases only due to missing acquisition of baseline data during external medical treatment. The median score for the HCT-CI was 0 for TEAM and 1 for Treo/Flu (*p* = 0.15; Mann–Whitney *U* test). Before auto-HSCT 23/36 (63.9%), 4/5 (80.0%) and 4/16 (25.0%) patients with DLBCL, FL, and MCL suffered from relapse and 13/36 (36.1%), 1/5 (20.0%), and 1/16 (6.3%) were classified as refractory, respectively. The majority of patients with MCL 11/16 (68.8%) were primarily intended to receive auto-HSCT. Patients in the TEAM group were significantly younger with a median age of 54 years (range, 23–63 years) versus 65 years (range, 49–73 years) in the Treo/Flu group (*p* < 0.0001; Mann–Whitney *U* test). The median number of pretreatment lines were 3 for TEAM and 2 for Treo/Flu, respectively (*p* = 0.26; Mann–Whitney *U* test). At the time of auto-HSCT, 8/35 (22.9%) of patients conditioned with TEAM and 4/22 (18.2%) with Treo/Flu conditioning were in CR, 23/35 (65.7%) and 16/22 (72.7%) had partial response (PR) while 4/35 (11.4%), and 2/22 (9.1%) suffered from progressive disease (PD), respectively (*p* = 0.75, CR vs. non-CR, Fisher’s exact test). The median follow-up of patients after auto-HSCT was 1.61 years (range, 0.18–7.96 years) for TEAM and 1.85 years (range, 0.25–4.87 years) for Treo/Flu (*p* = 0.77, Mann–Whitney *U* test).

### Adverse events

Infectious and non-infectious adverse events were reported for all patients receiving auto-HSCT. While the majority of patients suffered from therapy associated Grade III-IV mucositis and stomatitis (24/35, 68.6%) as well as infectious complications (34/35, 97.1%) after TEAM, these were less frequently observed after Treo/Flu conditioning (1/22, 4.5%, *p* < 0.0001 and 16/22, 72.7%, *p* < 0.0105; all Fisher’s exact test), respectively. A detailed breakdown of recorded toxicities is figured in Table 2.

**Table 2:** Safety data of HDT.

Adverse event according to CTCAE version 5.0	TEAM (*n* = 35)	Treo/Flu (*n* = 22)	*P*-value [Fisher’s exact test]
Diarrhea			
Grade I-II	4 (11.4%)	3 (13.6%)	1.0000
Grade III-IV	9 (25.7%)	2 (9.1%)	0.1740
Infectious complications			
Grade I-II	0 (0%)	0 (0%)	1.0000
Grade III-IV	34 (97.1%)	16 (72.7%)	**0.0105**
Nausea and vomiting			
Grade I-II	3 (8.6%)	1 (4.5%)	1.0000
Grade III-IV	11 (31.4%)	7 (31.8%)	1.0000
Oral mucositis and stomatitis			
Grade I-II	2 (5.7%)	4 (18.1%)	0.1921
Grade III-IV	24 (68.6%)	1 (4.5%)	**<0.0001**
Skin toxicity			
Grade I-II	1 (2.9%)	4 (18.1%)	0.0674
Grade III-IV	2 (5.7%)	2 (9.1%)	0.6355
Other, non-infectious complications, any grade			
Cardiac arrhythmia	3 (8.6%)	1 (4.5%)	1.0000
Hemorrhagic cystitis	1 (2.9%)	0 (0%)	1.0000
Hepatic toxicity, hepatitis	1 (2.9%)	0 (0%)	1.0000
Renal toxicity, kidney failure	0 (0%)	0 (0%)	1.0000
Pulmonal toxicity	1 (2.9%)	0 (0%)	1.0000
Thrombosis	0 (0%)	1 (4.5%)	0.3360
Secondary malignancies	0 (0%)	2 (9.1%)	0.1447

*CTCAE* Common terminology criteria for adverse events NCI, Bethesda, MD, USA; figures in bold indicate significant *p*-values.

### Engraftment, transfusion support, and period of hospitalization

Following conditioning with TEAM and Treo/Flu, engraftment occurred after a median time of 19.0 days (range, 14–83 days) and 18.0 days (range, 10–32 days) for thrombocytes (*p* = 0.0979). Leukocytes engrafted within 10 days in median (TEAM: range, 9–16 days; Treo/Flu: range, 8–16 days; *p* = 0.0197). While there was no difference regarding transfusion of red blood cells (in median 5 versus 4 erythrocyte concentrates following TEAM and Treo/Flu, respectively; *p* = 0.13), patients after TEAM conditioning received significantly more transfusions of thrombocyte concentrates in comparison to patients after conditioning with Treo/Flu (in median 5 versus 4 thrombocyte concentrates, *p* = 0.0082). Regardless of associated side effects, patients were discharged from hospital following reinfusion of stem cells after a median of 20 days (range, 13–52 days) and significantly later after conditioning with TEAM in comparison to 17.5 days (range, 13–35 days) after Treo/Flu, respectively, (*p* = 0.0484; all Mann–Whitney *U* test).

### Outcome and survival

**Fig. 1:**
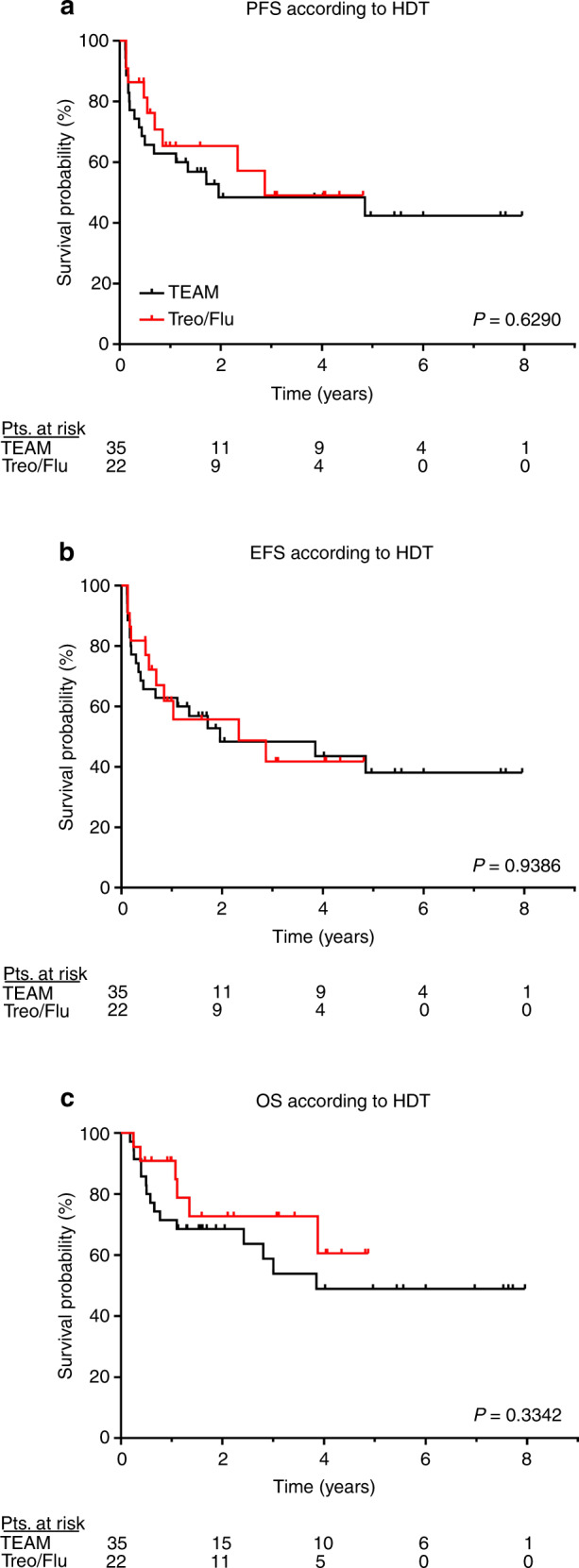
Outcomes of autologous stem cell transplantation after conditioning with TEAM (black line) or Treo/Flu (red line) in 57 patientes with R/R lymphoma. **a** Progression free survival (PFS), **b** Event free survival (EFS), and **c** Overall survival (OS). HDT High-dose chemotherapy, TEAM Myeloablative conditioning, Treo/Flu Reduced-intensity conditioning, figures in bold indicate significant *p*-values.

PFS and EFS were not significantly different between TEAM and Treo/Flu groups (median 1.96 vs. 2.87 years, *p* = 0.63, and median 1.96 vs. 2.33 years, *p* = 0.94), respectively. The median OS was 3.85 years for TEAM and not reached for Treo/Flu (*p* = 0.33). Differences were not significant in the comparison of high- (HML, i.e., DLBCL) or low-malignant lymphoma (LML, i.e., FL and MCL) subgroups regarding conditioning therapy (Supplementary Fig. S1; PFS: HML, *p* = 0.33 and LML, *p* = 0.55; EFS: HML, *p* = 0.79 and LML, *p* = 0.75; OS: HML, *p* = 0.34 and LML, *p* = 0.73, all Log-rank test). The 1-year PFS, EFS, and OS were estimated to be 62.9%, 62.9%, and 71.4% for TEAM and 65.3%, 61.9%, and 90.9% for Treo/Flu, respectively. After 2-years, PFS, EFS, and OS were 48.8%, 48.4%, and 68.6% for TEAM and 65.3%, 55.7%, and 72.7% for Treo/Flu, respectively (Fig. 1). At day +100 following auto-HSCT, the overall response rates were 77.1% (27/35) after conditioning with TEAM and 86.4% (19/22) after Treo/Flu, respectively.

The causes of death of four patients who died before day +100 following auto-HSCT were as follows: TEAM conditioning: sepsis; sepsis and pneumonia; sepsis, kidney failure, meningitis and progression; Treo/Flu conditioning: progression. Of those eight patients who additionally died before day +365 following auto-HSCT causes of death were as follows: sepsis and pulmonary failure; progression and kidney failure (1 patient, each); sepsis and liver failure (2 patients); and progression (3 patients) after TEAM conditioning and sepsis after surgery for endometrial carcinoma (1 patient) after Treo/Flu conditioning. Cumulative incidences of NRM after conditioning with TEAM and Treo/Flu were 4.7% (2/43) and 0% (0/22) at day + 100 (*p* = 0.26; Gray’s test) and 9.3% (4/43) versus 4.5% (1/22) at 1 year after auto-HSCT (*p* = 0.38; Gray’s test), respectively.

## Discussion

Although the treatment of patients with R/R lymphoma remains a challenge, HDT followed by auto-HSCT is an established treatment in this clinical situation [3, 31–34]. Conditioning with BEAM or TEAM are often used regimens but accompanied by a relevant toxicity [7, 9, 35]. Therefore, a lot of attention has been devoted to depict and to improve the conditioning regimens impact on HSCT outcome [36]. Aiming to extend the treatments tolerability for older or comorbid patients and to lower NRM, RTC regimens combining treosulfan with fludarabine, have been successfully introduced in the allogeneic setting [19, 20, 37]. None severe dose-limiting toxicities affecting lung, liver, heart, kidney or central nervous system were observed after treosulfan based conditioning [12, 14, 38]. The maximum tolerable dose of treosulfan is supposed to be 10 g/m² without following stem cell support and 47 g/m² with subsequent HSCT, respectively [39].

In analogy to this, we employed the Treo/Flu conditioning regimen followed by auto-HSCT for elderly and comorbid patients with NHL not eligible for intensive conditioning with TEAM from the clinicians’ perspective. Here we demonstrate the lack of significant difference of Treo/Flu in comparison to TEAM conditioning with regard to PFS, EFS, and OS. However, the median PFS and EFS after TEAM tended to be longer. Nevertheless, these results have to be interpreted against the background of the relatively short follow-up time and the small sample size. One additional reason could be the lower intensity of Treo/Flu in comparison to TEAM conditioning, what might be additionally mirrored in a shorter period of hospitalization and a lower dependency on transfusion support with thrombocytes. Applying the transplant conditioning intensity (TCI) score proposed by Spyridonidis et al., Treo/Flu (TCI _Treo/Flu_ = 3.5) has to be classified as intermediate intensity protocol [TCI 2.5–3.5] and TEAM (TCI _TEAM_ = 4.5) as high-risk [TCI 4–6] schemes [40]. Of note, in patients with HML the median PFS was not reached for Treo/Flu, while TEAM showed a better but once again nonsignificant PFS in patients with LML (Supplementary Fig. S1). The NRM at 1 year in our cohort insignificantly varied between 4.5% for the Treo/Flu-group and 9.3% for TEAM which is in line with published literature [9, 41–43].

The conditioning with Treo/Flu preceding auto-HSCT seems to be feasible in elderly patients which will sustain less complications. Severe oral mucositis developing after auto-HSCT is associated with an increased risk of duration of pain score ≥4, opioid use, dysphagia score ≥4, total parenteral nutrition, incidence and/or duration of fever and infection as well as duration of antibiotic use [44]. Patients receiving Treo/Flu suffered significant less frequently from Grade III-IV mucositis and stomatitis (*p* < 0.0001) as well as infectious complications (*p* = 0.0105; all Fisher’s exact test) if compared with patients after conditioning with TEAM, respectively. Of note, although the rate of infections following Treo/Flu tended to be lower in comparison to TEAM treated patients, it is comparable to those of former allogenic reports [12–14]. Tiothepa-induced cutaneous toxicity is a well-known adverse reaction and is also common in high proportions of treated patients [45]. Although the rate of Grade III-IV skin toxicities in this study was low, it is in line with the results of Sellner and colleagues [9]. However, we are meticulously paying attention to patients iterating body washes and daily changing of clothing. Finally, especially reported Grade I-II toxicities have to be interpreted with caution as they might follow a subclinical or asymptomatic course.

Further drawbacks of the present study are the retrospective character, the small sample size and imbalances concerning comparability of the groups, which allow only a limited interpretation of the results. Both groups varied significantly in age, with in median 11 years younger patients in the TEAM group. As nearly one out of four patients were initially diagnosed in an outward hospital or without appropriate scoring, robust prognostication using IPI, FLIPI and MIPI score failed. However, available scores are listed in Table 1. Although, the allocation of patients to conditioning protocol was based on attending physician’s choice and on the patient’s comorbidities, there were no significant differences in HCT-CI between both groups. This may be explained by the short time range over which the HCT-CI is normally assessed (days −40/−24 to −10 before HSCT) [46]. Due to the small numbers of patients, analysis of subgroups, e.g., with regard to primary refractory disease or early disease relapse were not performed.

This study is the first to present the efficacy, feasibility and safety of conditioning with Treo/Flu preceding auto-HSCT in patients with R/R lymphoma. It may offer a promising alternative to standard conditioning regimens in elderly patients, but finally, large, prospective, and randomized controlled trials with longer follow-up periods are required to rule out aforementioned inaccuracies and to verify our findings.

## Supplementary information

Supplemental Material

## Data Availability

The authors confirm that the data supporting the findings of this study are available within the article.
