# Supplementary material for: Treosulfan plus fludarabine versus TEAM as conditioning treatment before autologous stem cell transplantation for B-cell Non-Hodgkin lymphoma
Source: Bone Marrow Transplant. 2022 May 10;57(7):1164–70. doi: 10.1038/s41409-022-01701-x (PMC9262709; doi:10.1038/s41409-022-01701-x)
Supplement: Supplementary file 1 — Supplemental Material [file 41409_2022_1701_MOESM1_ESM.docx]

**Supplemental Material**

**Treosulfan plus fludarabine versus TEAM as conditioning treatment before** **autologous stem cell transplantation for B-cell Non-Hodgkin lymphoma**

Jochen J Frietsch ^1^, Jenny Miethke ^1^, Paul Linke ^1^, Carl C Crodel ^1^, Ulf Schnetzke ^1^, Sebastian Scholl ^1^, Andreas Hochhaus ^1^, Inken Hilgendorf ^1^

^1^ Klinik für Innere Medizin II, Hämatologie und internistische Onkologie, Universitätsklinikum Jena, Jena, Germany

Corresponding author:

Dr. med. Jochen Frietsch

Universitätsklinikum Jena, Abteilung Hämatologie und Internistische Onkologie

Am Klinikum 1, 07747 Jena, Germany

Phone: +49 (0) 36 41 / 9-32 42 79

Fax: +49 (0) 36 41 / 9-32 42 02

jochen.frietsch@med.uni-jena.de

ORCID ID <https://orcid.org/0000-0002-1476-8059>


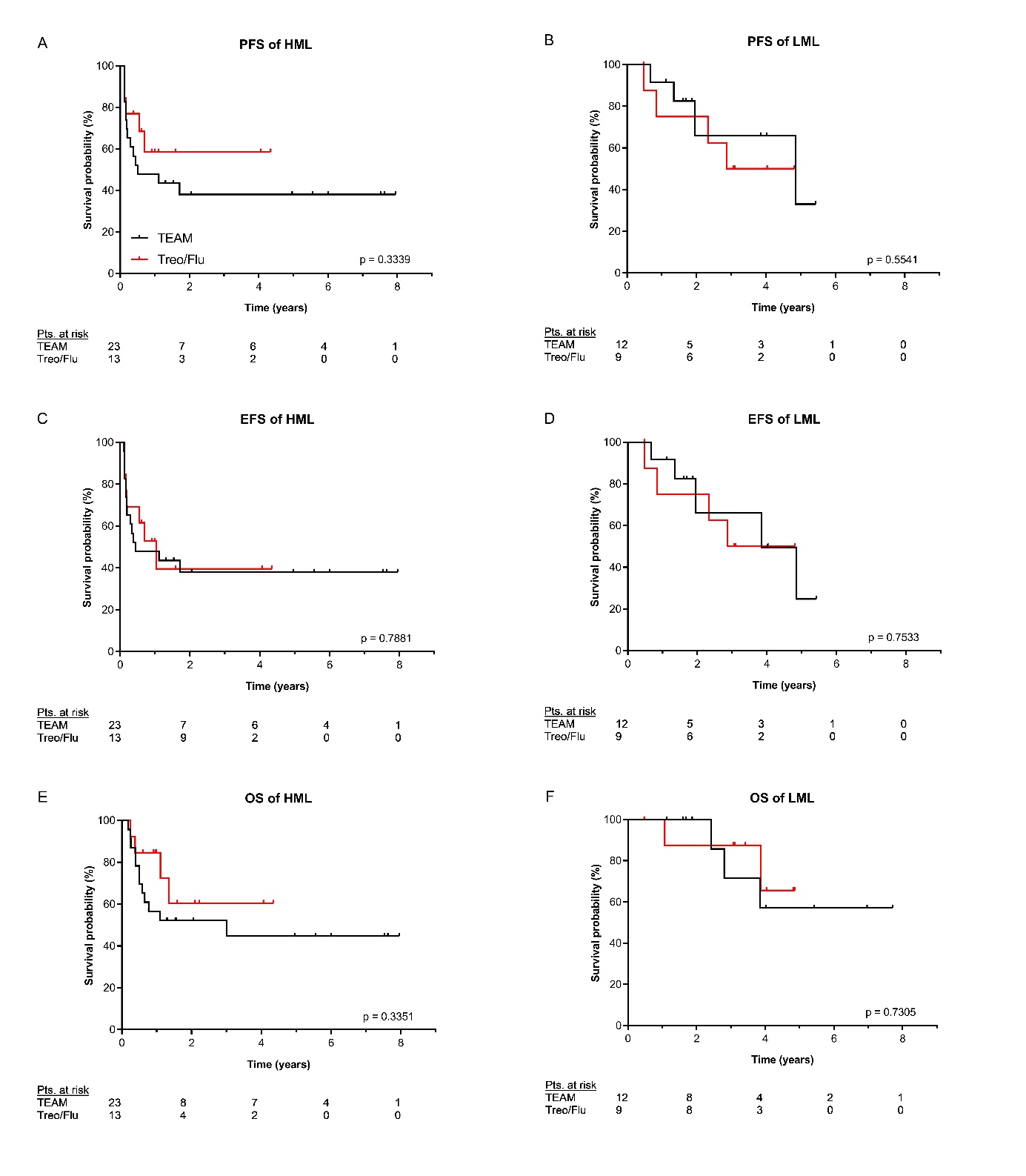
**Supplemental Figure S1: Outcome of disease subgroups according to HDT (A-F).** PFS, EFS and OS of patients with HML (A, C, E) and with LML (B, D, F), respectively.

EFS: event free survival; HDT: high-dose chemotherapy; TEAM: myeloablative conditioning; OS: overall survival; PFS: progression free survival; Treo/Flu: reduced-intensity conditioning; figures in bold indicate significant p-values
